# Social Cohesion, Connection and Prescription: Good ways for Preventing Suicide?

**DOI:** 10.1192/j.eurpsy.2022.127

**Published:** 2022-09-01

**Authors:** P. Courtet

**Affiliations:** University Hospital Montpellier, Emergency Psychiatry And Acute Care, Montpellier, France

**Keywords:** Suicide, Neuroimaging, pain, social connection

## Abstract

Despite the multiplication of the therapeutic strategies for psychiatric disorders during the last decades, the incidence of suicide has not decreased substantially. Specifically, the most commonly used strategy, which is the use of antidepressant treatments, is clearly nonsufficient to attain the goal of a substantial reduction of suicides. Then, we will discuss the hypothesis that people who are more sensitive to experiences of social disconnection may activate a pathophysiological process which lead to a greater risk of suicide, and by such its study may offer new avenues for assessing the suicide risk as well as in providing new therapeutic targets. It is well demonstrated that both the objective condition of being alone and loneliness are strongly associated with suicide. From a series of cognitive imaging studies, it appears that suicidal patients are more sensitive to social exclusion, relying on a neural network implicated in the pain matrix, and individuals experiencing more psychological pain in daily life would be less prone to activate orbitofrontal cortex in social contexts which would facilitate disadvantageous decision-making, leading to choose options (i.e. suicidal act) with short-term reward (i.e. relief from pain) in spite of the risks (i.e. death). As psychological pain predicts suicidal behaviour, suggesting the need in assessing it in clinical practice, in using alleviating drugs i.e. opiates or ketamine, and in implementing evidence-based psychosocial strategies, based on caring contacts and social prescription. In conclusion, there are many innovations available to help tackle social vulnerability, which should be a priority in suicide prevention.

**Disclosure:**

No significant relationships.

